# Integrated pulmonary index can predict respiratory compromise in high‐risk patients in the post‐anesthesia care unit: a prospective, observational study

**DOI:** 10.1186/s12871-021-01338-1

**Published:** 2021-04-21

**Authors:** Yasutoshi Kuroe, Yuko Mihara, Shuji Okahara, Kenzo Ishii, Tomoyuki Kanazawa, Hiroshi Morimatsu

**Affiliations:** 1 Department of Anesthesiology and Resuscitology, Graduate School of Medicine, Dentistry, and Pharmaceutical Sciences, Okayama University, 2-5-1 Shikata-cho, 700-8558 Kitaku, Okayama Japan; 2 Department of Anesthesiology and Oncological Pain Medicine, Fukuyama City Hospital, 5-23- 1 Zaocho, 721-8511 Hukuyama, Hiroshima Japan; 3 Department of Pediatric Anesthesiology, Okayama University Hospital, 2-5-1 Shikata-cho, 700-8558 Kitaku, Okayama Japan

**Keywords:** Integrated pulmonary index, Respiratory compromise, Post‐anesthesia care unit

## Abstract

**Background:**

Respiratory compromise (RC) including hypoxia and hypoventilation is likely to be missed in the postoperative period. Integrated pulmonary index (IPI) is a comprehensive respiratory parameter evaluating ventilation and oxygenation. It is calculated from four parameters: end-tidal carbon dioxide, respiratory rate, oxygen saturation measured by pulse oximetry (SpO_2_), and pulse rate. We hypothesized that IPI monitoring can help predict the occurrence of RC in patients at high-risk of hypoventilation in post-anesthesia care units (PACUs).

**Methods:**

This prospective observational study was conducted in two centers and included older adults (≥ 75-year-old) or obese (body mass index ≥ 28) patients who were at high-risk of hypoventilation. Monitoring was started on admission to the PACU after elective surgery under general anesthesia. We investigated the onset of RC defined as respiratory events with prolonged stay in the PACU or transfer to the intensive care units; airway narrowing, hypoxemia, hypercapnia, wheezing, apnea, and any other events that were judged to require interventions. We evaluated the relationship between several initial parameters in the PACU and the occurrence of RC. Additionally, we analyzed the relationship between IPI fluctuation during PACU stay and the occurrences of RC using individual standard deviations of the IPI every five minutes (IPI-SDs).

**Results:**

In total, 288 patients were included (199 elderly, 66 obese, and 23 elderly and obese). Among them, 18 patients (6.3 %) developed RC. The initial IPI and SpO_2_ values in the PACU in the RC group were significantly lower than those in the non-RC group (6.7 ± 2.5 vs. 9.0 ± 1.3, *p* < 0.001 and 95.9 ± 4.2 % vs. 98.3 ± 1.9 %, *p* = 0.040, respectively). We used the area under the receiver operating characteristic curves (AUC) to evaluate their ability to predict RC. The AUCs of the IPI and SpO_2_ were 0.80 (0.69–0.91) and 0.64 (0.48–0.80), respectively. The IPI-SD, evaluating fluctuation, was significantly greater in the RC group than in the non-RC group (1.47 ± 0.74 vs. 0.93 ± 0.74, *p* = 0.002).

**Conclusions:**

Our study showed that low value of the initial IPI and the fluctuating IPI after admission to the PACU predict the occurrence of RC. The IPI might be useful for respiratory monitoring in PACUs and ICUs after general anesthesia.

## Background

Postoperative pulmonary complications are common and crucial events because they significantly increase the morbidity, mortality, the lengths of intensive care unit and hospital stay, and the healthcare costs [1–3]. Particularly in the post-anesthesia care unit (PACU), severe and preventable respiratory events may occur frequently [4, 5]; these events were defined as respiratory compromise (RC) [6]. Thus, it is important to predict and prevent these events in PACUs to improve patients’ outcomes.

To monitor respiration, it is important to monitor oxygenation and ventilation [7]. Currently, respiratory monitoring in PACUs is performed only using oxygen saturation measured by pulse oximetry (SpO_2_). One of the methods for monitoring ventilation is capnography. However, although it has become an integral part of anesthesia in the operating room for more than 30 years, its value beyond these confinements is limited [8].

The integrated pulmonary index (IPI) is a newly developed index for respiratory monitoring. It is calculated automatically from four components using a fuzzy logic model—end tidal carbon dioxide (ETCO_2_), respiratory rate (RR), SpO_2_, and pulse rate—and evaluated on a 10-point scale; scores ≥ 8 points are within normal range and those ≤ 4 points suggest requirement of interventions [9]. The IPI algorithm summarizes the state of ventilation and oxygenation at the point in time. Previous studies reported that IPI correlated with respiratory physiological parameters of patients undergoing sedation for surgeries or for colonoscopy [10, 11]. However, there is limited evidence on its effectiveness and usefulness in other clinical situations including postoperative setting.

The purpose of this study was to evaluate the clinical relevance of the IPI and its relationships with postoperative RC. We hypothesized that the IPI could be useful for predicting RC in high-risk patients in PACUs.

## Methods

### Ethical considerations

The study was approved by the institutional ethics review boards of both participating hospitals (No. 2135; No. 205). All patients provided written informed consent prior to inclusion in the study. This manuscript adheres to the applicable Strengthening the Reporting of Observational Studies in Epidemiology guidelines [12].

### Study design and patients

This was a prospective, observational, two-center study conducted in the PACUs of Okayama University Hospital and Fukuyama City Hospital in Japan from October 2014 to March 2015.

We enrolled patients who were scheduled for admission to the PACUs after elective surgery under general anesthesia and were at high risk of postoperative hypoventilation. The criteria for high-risk patients were older age (≥ 75-year-old) or obesity (body mass index ≥ 28). The exclusion criteria were as follows: (1) age < 18 years, (2) ambulatory surgery. Patients undergoing surgery such as craniotomy, thoracotomy, cardiac surgery were scheduled for transfer to intensive care units without admission to PACUs.

We screened eligible patients before surgery and obtained informed consents. After admission to the PACU, patients were monitored using Capnostream™ 20P® (Medtronic, Boulder, CO) for more than 30 min in addition to the standard monitors, and any respiratory events and interventions were recorded by the PACU nurses. Supplemental oxygen was administered to patients according to the usual standard clinical practice at the institution.

### Variables

Expired gas sampling lines were attached to extubated patients upon admission to the PACU and the initial ETCO_2_, RR, SpO_2_, pulse rate, and IPI values were recorded. These parameters were measured until patients were transferred out of the PACU using Capnostream™ 20P®. The sampling line of this device features oral and nasal sampling as well as a supplemental oxygen delivery system [13]; it has a small mouth and nose cover to catch exhaled gas and has apertures for oxygen delivery. The device measures the ETCO_2_ and RR by sampling exhaled gas and the SpO_2_ and pulse rate by pulse oximetry. Furthermore, the IPI is calculated automatically from four parameters and all values are displayed on a screen. The calculation methods use fuzzy logic inference model based on expert clinical opinions. After the provisional IPI is assigned according to the matrix table of RR and ETCO_2_, the definite IPI is decided finally adding evaluation of SpO_2_ and PR. This algorithm was verified by comparison to experts’ scoring of clinical scenarios [9].

If RC would occur, anesthesiologists or nurses recorded the time of the occurrence and the details of the RC in the medical records. Patients’ characteristics, including age, sex, body mass index, American Society of Anesthesiologists physical status, surgical procedure type, anesthesia time, and surgery time were retrieved from the electronic anesthetic records.

### Outcomes

The primary outcome was the occurrence of RC in the PACU. We defined RC as any respiratory event resulting in prolonged PACU stay or transfer to the intensive care unit, such as airway narrowing, hypoxemia (SpO_2_ < 92 %), hypercapnia (partial pressure of carbon dioxide in arterial blood [PaCO_2_]. > 45mmHg and pH < 7.35), wheezing, apnea, and any other events that were judged to require interventions by anesthesiologists or nurses. To evaluate the respiratory status stability, we selected the IPI fluctuations during the stay in the PACU. Specifically, we recorded the IPI values every 5 min within an hour in each patient and evaluated them as standard deviations (SDs) of the IPI (IPI-SDs). After patients were transferred out of the PACU, we extracted the data from the device on a universal serial bus; the day and time, SpO_2_, ETCO_2_, RR, pulse rate, and IPI. In cases of data loss because the saturation probe or gas sampling cannula had been dislocated, removed, or not connected to the Capnostream™ 20P®, the patients were excluded from the analysis. If RC had occurred, we obtained the details from the medical records.

### Statistical analysis

The study population was divided into two groups according to the occurrence of RC: RC group and non-RC group. We compared the initial parameters at admission to the PACU between the two groups using Wilcoxon’s rank-sum test to identify the predictors of RC occurrence.

To evaluate the IPI fluctuation after admission to the PACU, we used the individual IPI-SDs of each patient. Next, we calculated the mean IPI-SDs of both groups and compared them using Wilcoxon’s rank-sum test.

Data were presented as absolute values (%), medians (interquartile range), or means ± SDs. A p value < 0.05 was considered statistically significant in all analyzes.

## Results

Overall, 4,159 patients underwent surgery under general anesthesia during the study period. Of these, 2,621 patients were admitted to the PACUs. Among them, 291 patients (11.1 %) fulfilled at least one of the criteria of this study. However, three patients were excluded due to missing data. Consequently, 288 patients (199 elderly, 66 obese, and 23 elderly and obese patients) were included in this study analysis. The baseline demographic and clinical characteristics of the patients are shown in Table 1. The mean age was 74.8 ± 14.2 years and the mean body mass index was 25.0 ± 5.2. The mean anesthesia time was 169.4 ± 95.2 min. According to the surgery type, patients undergoing orthopedic (25.7 %), and abdominal (21.2 %) surgery comprised the highest proportion.

**Table 1:** Baseline demographic and clinical characteristics

Variables	Total (*n* = 288)	RC group (*n* = 18)	Non-RC group (*n* = 270)
Age	74.8 ± 14.2	76.3 ± 11.8	74.6 ± 14.4
Sex (Male) (%)	43.4	44.4	43.3
Body mass index	25.0 ± 5.2	27.3 ± 6.5	24.9 ± 5.1
ASA-PS	2 [2-3]	2 [2-3]	2 [2-3]
Anesthesia time (min)	169 ± 95	207 ± 110	167 ± 94
Surgical Time (min)	121 ± 82	154 ± 99	118 ± 80
Type of surgery			
Orthopedic	74 (25.7%)	4 (22.2 %)	70 (25.9%)
Abdominal	61 (21.2%)	4 (22.2%)	57 (21.1%)
Urologic	38 (13.2%)	2 (11.1%)	36 (13.3%)
Otorhinolaryngologic	29 (10.1%)	4 (22.2%)	25 (9.3%)
Breast internal secretion	28 (9.7%)	2 (11.1%)	26 (9.6%)
Obstetrics and gynecology	15 (5.2%)	1 (5.6%)	14 (5.2%)
Other	43 (14.9%)	1 (5.6%)	42 (15.6%)

All values reported as n (%), mean ± standard deviation, or median [interquartile range]

*RC* respiratory compromise; *ASA-PS* American Society of Anesthesiologists physical status

### Outcomes

Among the 288 patients, 18 patients (6.3 %) developed RC during their PACU stay. The most frequent cause of RC was hypoxia, which occurred in seven patients (38.9 %). Airway narrowing occurred in three, apnea in three, hypercapnia in one, wheezing in one, and other respiratory events occurred in three patients (Table 2). Most cases of RC occurred within 30 min after admission to the PACU. The incidence of RC was 5.9 % in elderly patients and 9.0 % in obese patients. The length of PACU stay of patients with RC was longer than that of patients without RC (101 ± 48 min versus 61 ± 30 min, *p* < 0.001).

**Table 2:** Types of respiratory compromise

Respiratory compromise	N (%)
Hypoxemia	7 (38.9)
Airway narrowing	3 (16.7)
Apnea	3 (16.7)
Hypercapnia	1 (5.6)
Wheezing	1 (5.6)
Other	3 (16.7)
‒ Insufficient expectoration of sputum	1 (5.6)
‒ Rapid shallow breathing	1 (5.6)
‒ Respiratory alkalosis	1 (5.6)

### Association between RC and the initial parameters

The comparison of the initial parameters on admission to the PACU between the RC and non-RC groups is presented in Table 3. The mean initial IPI of the RC group was significantly lower than that of the non-RC group (6.7 ± 2.5 versus 9.0 ± 1.3; *p* < 0.001). The mean initial SpO_2_ of the RC group was also significantly lower than that of the non-RC group (95.9 ± 4.2 % versus 98.3 ± 1.9 %; *p* = 0.040). In contrast, there were no significant differences in the mean ETCO_2_, RR, and pulse rate between the two groups.

**Table 3:** The relationship between parameters at admission in PACU and the incidence of respiratory compromise

Parameters at admission in PACU	RC group	Non-RC group	*p*-value
Integrated pulmonary index	6.7 ± 2.5	9.0 ± 1.3	< 0.001
SpO_2_	95.9 ± 4.2	98.3 ± 1.9	0.04
ETCO_2_	37.6 ± 11.9	38.6 ± 6.26	0.94
RR	14.2 ± 6.0	13.6 ± 4.1	0.70

All values reported as mean ± standard deviation

*PACU *post-anesthesia care unit; *RC* respiratory compromise; *SpO*_2_ oxyhemoglobin saturation measured by pulse oximetry; *ETCO*_2_ end-tidal carbon dioxide; *RR* respiratory rate

**Fig. 1:**
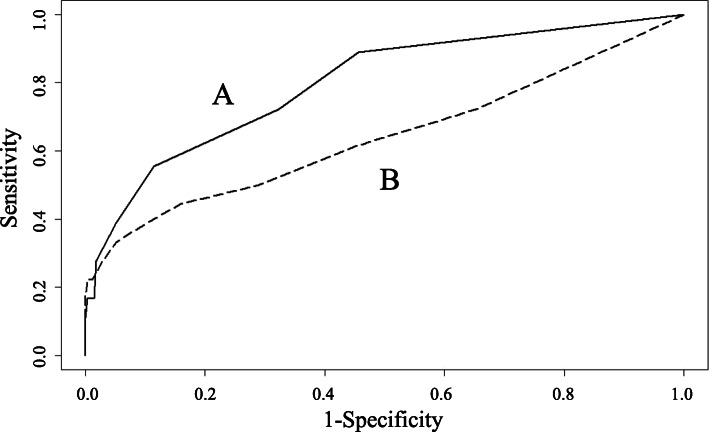
Predictors of RC in PACUs. Comparisons of the receiver operating characteristic curves for the initial IPI (A) and SpO_2_ (B) values as predictors of RC in PACUs. The AUCs of the IPI and SpO_2_ were 0.80 and 0.64, respectively. RC: respiratory compromise, PACU: post-anesthesia care unit, IPI: integrated pulmonary index, SpO_2_: oxyhemoglobin saturation measured by pulse oximetry

Following these results, receiver operating characteristic (ROC) curves were generated to calculate the area under the curve (AUC) for the significant initial parameters (Fig. 1). The AUCs for the initial IPI and SpO_2_ were 0.80 (95 % confidence interval [CI].: 0.69–0.91) and 0.64 (95 % CI: 0.48–0.80), respectively. When the cut-off point of the initial IPI was 7 to predict RC, its sensitivity, specificity, and likelihood ratio were 55.6 %, 88.5 % and 4.8, respectively. When the cut-off point of the initial SpO_2_ was 96 %, its sensitivity, specificity, and likelihood ratio were 44.4 %, 84.1 % and 2.8, respectively.

### IPI fluctuation after admission to the PACU

**Fig. 2:**
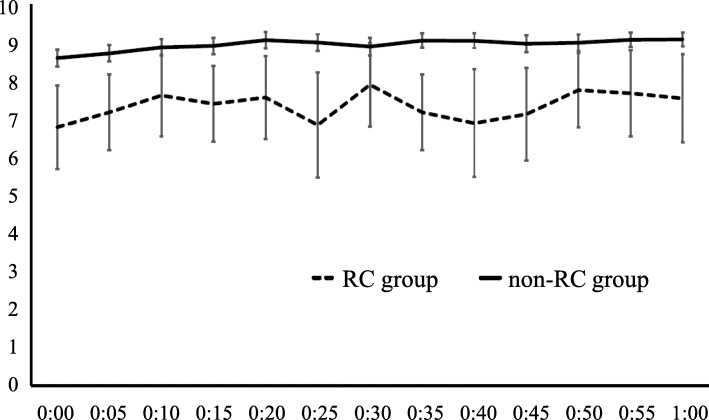
IPI fluctuation during PACU stay. This graph shows the fluctuation of the IPI values of each group at every 5 min within 1 h after admission to the PACU. The means and 95 % CIs of the IPI values at every point is described on the continuous line and the dashed line connects the mean IPI values for every 5 min. The ends of the upper and lower whiskers represent the 95 % CIs. IPI: integrated pulmonary index, PACU: post-anesthesia care unit, CI: confidence interval, RC: respiratory compromise

In the analysis of IPI fluctuation after admission to the PACU, 20 patients were excluded because of missing data. Figure 2 displays the IPI value trends after admission to the PACU of both groups. In the non-RC group, the IPI values were stable at a high level (8−10 points); however, in the RC group, those values distributed in a relatively wide range (5−9 points). Furthermore, the mean IPI-SD in the RC group was significantly greater than that in the non-RC group (1.47 ± 0.74 versus 0.93 ± 0.74, *p* = 0.002), indicating that the IPI values fluctuated in a higher proportion of patients in the RC group compared to the non-RC group.

## Discussion

### Key results

In this study, we investigated whether the IPI can predict RC in high-risk adult patients after general anesthesia. We determined that 6.3 % of these patients developed RC and that their stay in PACUs was significantly prolonged. Patients with RC had lower IPI and SpO_2_ values on admission to the PACU than patients without RC. The AUCs for the IPI tended to be higher than that for SpO_2_, but not significant. After admission to the PACUs, the IPI in the RC group had significantly greater fluctuations than that in the non-RC group throughout the PACU stay.

### Relationship to previous findings

The incidence of RC was 6.3 % in our high-risk patients after surgeries under general　anesthesia, except cardiovascular, thoracic, and craniotomy surgeries, which require postoperative intensive care. Several studies have surveyed the incidence of postoperative RC in the PACU; in these studies, the incidence ranged from 1.3 to 16 % [4, 14, 15]. We attributed the difference with our findings to the difference in the definition of respiratory events and the inclusion criteria among the studies. In our study, we determined the definition of RC in reference to previous studies and included only high-risk patients [11].

In present study, the initial values of the IPI on admission to the PACU could be a predictor of the occurrence of RC in PACUs. Although we could not show the superiority of the prediction ability of the IPI to that of the SpO_2_, there are some studies that show the importance of evaluating the ventilation in addition to the oxygenation.

Thomas et al. reported that capnography, but not pulse oximetry, alerted impending respiratory depression in their study including postoperative patients receiving patient-controlled analgesia [16]. Kaur et al. verified on the role of the IPI in identifying extubation failure. The IPI value 1 h after extubation was significantly lower in the failed extubation group than in the successful extubation group [17]. These reports support the possibility of IPI as a respiratory monitoring tool in the perioperative period including PACU.

Additionally, greater fluctuations of the IPI values could indicate risk of RC. Not only the initial IPI value, but its fluctuations should be monitored as well, as respiratory status instability is a risk for RC. Lynn et al. reported that repetitive reductions in airflow and SpO_2_ were followed by arousal failure and hypoxic death [7]. However, there has been no study that statistically analyzed the degree of respiratory status fluctuation after general anesthesia. As the perioperative patients’ respiratory status can be continuously monitored using capnography in addition to SpO_2_, it would detect RC more preciously to follow up the fluctuation of the IPI values.

### Clinical implications

Capnometer is commonly used for intubated patients during surgery, but our study presented that ETCO_2_ can be measured noninvasively using nasal cannula with sampling line and IPI was evaluable for non-intubated patients. For high-risk patients such as patients with obstructive sleep apnea symptoms, [18] comprehensive oxygenation and ventilation monitoring would enable early recognition and treatment of RC. As IPI classifies patient status on simple 10-point scale (≥ 8: within normal range and ≤4: requirement of interventions), it would particularly help junior doctors or co-medical staffs in PACU to grasp respiratory conditions objectively regardless of their experiences and knowledge. Large-scale prospective studies will be required to assess the usefulness of IPI algorithm as an early warning tool under these situations.

### Limitations

The limitations of this study are as follows. First, our study was not blinded. All patients received routine care by the PACU staff, mainly nurses or anesthesiologists, and the staff were not blinded to additional parameters (IPI, ETCO_2_, and RR) displayed by the Capnostream™ 20P®. This could have affected the predictive value of these parameters. However, most of the staff were not familiar with the IPI and we informed only some of the staff members of the related details. Second, the time to the occurrence of RC was on average only 30 min. However, we can add detailed physiological and laboratory measures with increased nursing ratio or X-ray, if required. Third, we enrolled only patients who were elderly or obese as a high-risk group in this study; hence, our findings cannot be generalized to all patients. Finally, the incidence of RC could have been influenced by our definition. Because there is no clear definition, we determined the definition of RC referring to previous studies. We believe that the incidence of RC in our study was quite reasonable.

## Conclusions

Our results demonstrated that the IPI can predict the occurrence of RC in high-risk patients in PACUs. Therefore, evaluation of the IPI, including the SpO_2_, RR, ETCO_2_, and pulse rate, might be useful for respiratory monitoring at PACUs and intensive care units after general anesthesia.

## Data Availability

The datasets generated and analyzed during the present study are available from the corresponding author on reasonable request.

## Abbreviations

AUC: Area under the curve
CI: Confidence interval
ETCO_2_: End tidal carbon dioxide
IPI: Integrated pulmonary index
IPI-SD: Standard deviation of the IPI every five minutes
PaCO_2_: Partial pressure of carbon dioxide in arterial blood
PACU: Post-anesthesia care unit
RC: Respiratory compromise
RR: Respiratory rate
SD: Standard deviation
SpO_2_: Oxygen saturation measured by pulse oximetry
